# MicroRNA Isolation by Trizol-Based Method and Its Stability in Stored Serum and cDNA Derivatives

**DOI:** 10.31557/APJCP.2019.20.6.1641

**Published:** 2019

**Authors:** Keson Trakunram, Nidanut Champoochana, Pichitpon Chaniad, Paramee Thongsuksai, Pritsana Raungrut

**Affiliations:** 1 *Department of Biomedical Sciences, *; 2 *Department of Pathology, Faculty of Medicine, Prince of Songkla University, Songkhla, Thailand. *

**Keywords:** Trizol, based method, stability, serum, miRNA, cDNA derivatives

## Abstract

MicroRNAs (miRNAs) are small, non-coding RNA molecules that regulate gene expression at the post-transcriptional level. Since aberrant expression of miRNAs has been proposed as usage for blood-based biomarkers, hence reliable techniques for miRNA isolation as well as stability of miRNAs in various stored conditions needs to be explored. This present study aimed to investigate the efficacy of the Trizol-based isolation technique and the stability of miRNAs in stored serum and cDNA derivatives. Total RNA, including miRNAs, was isolated from human serum and a comparison of the efficiency of the Trizol^®^LS reagent isolation method against the miRNeasy^®^mini kit was conducted. Expression of RNU6, miR-145, and miR-20a was determined by quantitative real-time polymerase chain reaction (qRT-PCR). We showed that Trizol^®^LS isolation yielded significantly lower RNA concentrations than that of the miRNeasy^®^mini kit by approximately 35%. Purity of the isolated RNAs by both methods was similar. RNU6, miR-145, and miR-20a degraded at room temperature, but all genes were stable at 4ºC, -20ºC and -80ºC for a 72-hrs period, in both serum and cDNA storage conditions. In the stored cDNA derivatives, we observed the stability of RNU6, miR-145, and miR-20a for 3 months at -20ºC, and all genes also resisted 4 repeated freeze-thaw cycles at -20ºC. In conclusion, the Trizol-based method is efficient as well as economical to use for quantification of circulating miRNAs. In addition, we proposed that the storage of miRNA-derived cDNAs may be an alternative choice to avoid the stability effect.

## Introduction

MicroRNAs (miRNAs) are short, small non-coding RNAs of 19-22 nucleotides in length. They regulate gene expression at the post-transcriptional level through binding to the 3’-untranslated region of target mRNAs, leading to degradation or translational repression (Esquela-Kerscher and Slack, 2006). Currently, there are approximately 4,076 miRNAs having been identified in humans (miRTarbase 7.0, Release date 09/15/2017) (Chou et al., 2018), and these miRNAs regulate more than 60% of coding genes (Friedman et al., 2009). Accumulating studies have revealed that miRNAs play a crucial role in various biological processes such as; differentiation, proliferation, apoptosis, and metabolism (Calin and Croce, 2006; Bartel, 2009) in addition, they can be detected within body fluids, including; plasma, serum, saliva, sputum, and urine (Chen et al., 2008; Shen et al., 2011). As circualting miRNA levels, particularly in the blood, are found to be correlated with progression, therapeutic response, and survival of cancer patients (Chen et al., 2008; Rani et al., 2013; Li et al., 2015) the potentail use of miRNAs as non-invasive biomarkers is interesting. In the blood, more than 90% of all miRNAs are presented in vesicle-free miRNAs, which are associated with the Argonaute (Ago) protein (Turchinovich et al., 2011; Turchinovich et al., 2012). Due to their small size coupled with their attachment to the Ago protein, the study of miRNAs isolation considering both reliable and inexpensive technique as well as their stability under relevant clinical conditions is critical.

Recently, there have been several techniques for isolation of circulating miRNAs, including; Trizol^®^LS extraction based on liquid-liquid extraction and commercial-kit methods. Sourvinou et al., (2013) demonstrated that Trizol^®^LS extraction is less effective and reliable than the commercial kits (Sourvinou et al., 2013). In so saying, this study is in contrast with a study by McDonald et al., (2011) by showing that yields of miRNAs are increased by Trizol^®^LS extraction as compared to commercial miRNA isolation kits. Besides these miRNA isolation techniques, stability of the circulating miRNAs has shown considerable inconsistency between different studies. Several studies have revealed that the circulating miRNAs are presented in a highly stable manner. For example; miRNAs are stable at room temperature and 4°C at least 8 hrs unhemolyzed blood samples (Wu et al., 2016). Similarly, serum miRNAs are resistant to RNaseA digestion, low/high pH, repeated freeze-thaw cycles at -80°C, and stable at -20˚C and -80˚C for at least 4 years (Chen et al., 2008; Mitchell et al., 2008; Grasedieck et al., 2012). However, an increasing number of studies have demonstrated that the instability of the miRNAs has been found in various storage conditions. Degradation of miRNAs are obviously seen in stored serum, or plasma at room temperature within 5-24 hrs (Mitchell et al., 2008; Koberle et al., 2013; Wu et al., 2016; Glinge et al., 2017). In addition, the miRNAs are unstable when such samples are stored at 4°C, -20°C, and -80°C within different time periods (Sourvinou et al., 2013; Wu et al., 2016), and the repeated freeze-thaw cycles of these samples also leads to a significant decrease in miRNA levels (Grasedieck et al., 2012; Ge et al., 2014; Zhao et al., 2014; Glinge et al., 2017).

Despite the facts that circulating miRNAs present great potential as non-invasive biomarkers by a number of studies, an efficacy of extracted miRNAs by Trizol^®^LS method and miRNA stability in stored conditions are still ambiguous. The aim of this study was to evaluate the effect of miRNA isolation from serum using Trizol^®^LS reagent. In addition, we also investigated the effects of the storage of serum and cDNA derivatives in different storage conditions, as to the stability of miRNAs and RNU6, which is normally used as an internal control. 

## Materials and Methods

*Sample preparation*

The study protocol was approved by the Ethics Committee of the Faculty of Medicine, Prince of Songkla University (REC59-011-05-1). Three healthy Thai volunteers who were over the age of 25 years and had not previously been diagnosed with cancer were included in this study. All healthy volunteers had signed written consent, before blood sampling. Blood samples (5 ml each) were collected into clotting tubes (Greiner Bio-One, Kremsmünster, Austria) by venipuncture at Songklanagarind Hospital, Faculty of Medicine, Prince of Songkla University, Songkhla, Thailand and then kept at room temperature for 30 mins. Serum was isolated by centrifugation at 3,400xg for 10 mins at room temperature and then filtrated through a 0.22 µm pore size membrane filter (Merck Milipore, Darmstadt, Germany). Aliquots of these serum samples were removed, and frozen in 1.5 ml RNase-free tubes for further processing. 

*RNA isolation*

The total RNA was isolated from a 250 µl serum, and the extraction method was followed according to the manufacturer’s protocol of the miRNeasy^®^serum/plasma kit (Qiagen, Hilden, Germany). For the Trizol-based method, serum samples (50-250 µl) were added into 750 µl of Trizol^®^LS reagent (Invitrogen, California, USA). Mixtures were then gently inverted 5-8 times, and incubated at room temperature for 15 mins. After which, 200 µl of chloroform (J.T. Baker, Pennsylvania, USA) was added. Samples were mixed by inverting for 15 secs and once more incubated at room temperature for 5 mins. After, centrifugation at 12,000xg for 15 mins, at 4ºC, the upper aqueous phase was carefully transferred into a new tube, upon which 500µl isopropanol (J.T. Baker, Pennsylvania, USA) was added, and then incubated for 10 mins at room temperature, before then being centrifuged at 4ºC, 12,000xg for 10mins. Pellets were washed with 75% ethanol (J.T. Baker, Pennsylvania, USA), air-dried at room temperature for 30 mins, and re-suspended in 20 μl of RNase-free water (Qiagen, Hilden, Germany). Concentration and purity of the total RNA was assessed by NanoDropND-1000 UV-Vis Spectrophotometer (Thermo Scientific, Massachusetts, USA) at an optical density (OD) ratio of A260/280 nm and A260/230 nm.

*Reverse transcription and quantitative real-time polymerase chain reaction (qRT-PCR)*

miRNAs, and U6 small nuclear RNA (RNU6) were polyadenylated and converted into cDNA with oligo-dT primer via use of miScript II RT Kit (Qiagen, Hilden, Germany). In brief, 50 ng of total RNA was mixed with 4 μl miScriptHiFlex buffer, 2 μl miScript nucleics mix, and 2 μl miScript reverse transcriptase mix in a final volume of 20 µl. Reverse transcription reaction was performed at 37°C for 60 mins, and 95°C for 5 mins by a Thermal Cycler (Bio-Rad, California, USA). Then, the cDNA was diluted by adding 200 μl of RNase-free water. Expression of the miRNAs and RNU6 was quantified by BioRad CFX96 qPCR System (Bio-Rad, California, USA) using a miScript SYBR^®^ Green PCR kit (Qiagen, Hilden, Germany). Reaction was performed in the final volume of 25μl, containing 2.5 μl diluted cDNA, 12.5 μl SYBR green PCR master mix, 2.5 μl miScript universal primer, and 2.5 μl specific forward primers for RNU6, miR-145, and miR-20a (Qiagen, Hilden, Germany) in a final volume of 25 μl. Cycling conditions for qRT-PCR were as follows: 95ºC for 15 mins, followed by 50 cycles of 94ºC for 15 secs, 55.7ºC (RNU6), or 57ºC (miR-145), or 56.9ºC (miR-20a) for 30 secs, and 70ºC for 30 secs. Expression was presented as cycle threshold (Ct) values, which are the number of cycles required for the fluorescent signal to cross the threshold. Experiments were performed in triplicate, with the mean values for Ct values being used for the calculation. Sequences of mature miRNAs and RNU6 were shown in Table 1. 

*Experimental procedures*

*- Impact of storage conditions*

Serum was suddenly aliquoted, after blood processing as described above. Different aliquots of serum were stored at different temperatures (room temperature, 4ºC, -20ºC, and -80ºC) for different durations (0, 1, 3, 5, 24, 48, and 72 hrs), until extraction. Meanwhile, cDNA derivatives, which were immediately synthesized after total RNA isolation, were stored at different temperatures, and durations in the same way as the serum. Expression levels of each duration under each temperature were compared to a time of 0 hrs. 

In addition, to investigate the impact of intermediate-term storage of cDNA derivatives on miRNA stability, the cDNA derivatives were kept at -20ºC for 3 months. The expression levels at 3 months were compared to a time of 0 hrs.

*- Repetitive freeze-thaw cycles*

cDNAs, derived from miRNAs and RNU6, were aliquoted and frozen at -20ºC. Aliquot was then thawed at 4ºC, for approximately 1 hr, until completely thawed, then mixed properly with automatic pipettes. One to four, freeze–thaw cycles of cDNAs derivatives were performed, and each cycle was compared to a non freeze-thaw cycle.

*Statistical analysis*

Absolute Ct values were presented as mean ± standard deviation (SD) of two- or three- independent experiments. The differences between the expression level of each condition and controls were made using the independent student t-test with GraphPad Prism 5 (GraphPad Software Inc, California, USA). A P-value of less than 0.05 was considered as statistically significant difference.

## Results

*RNA isolation in serum samples by use of the Trizol* ^®^ *LS method*

**Figure 1:**
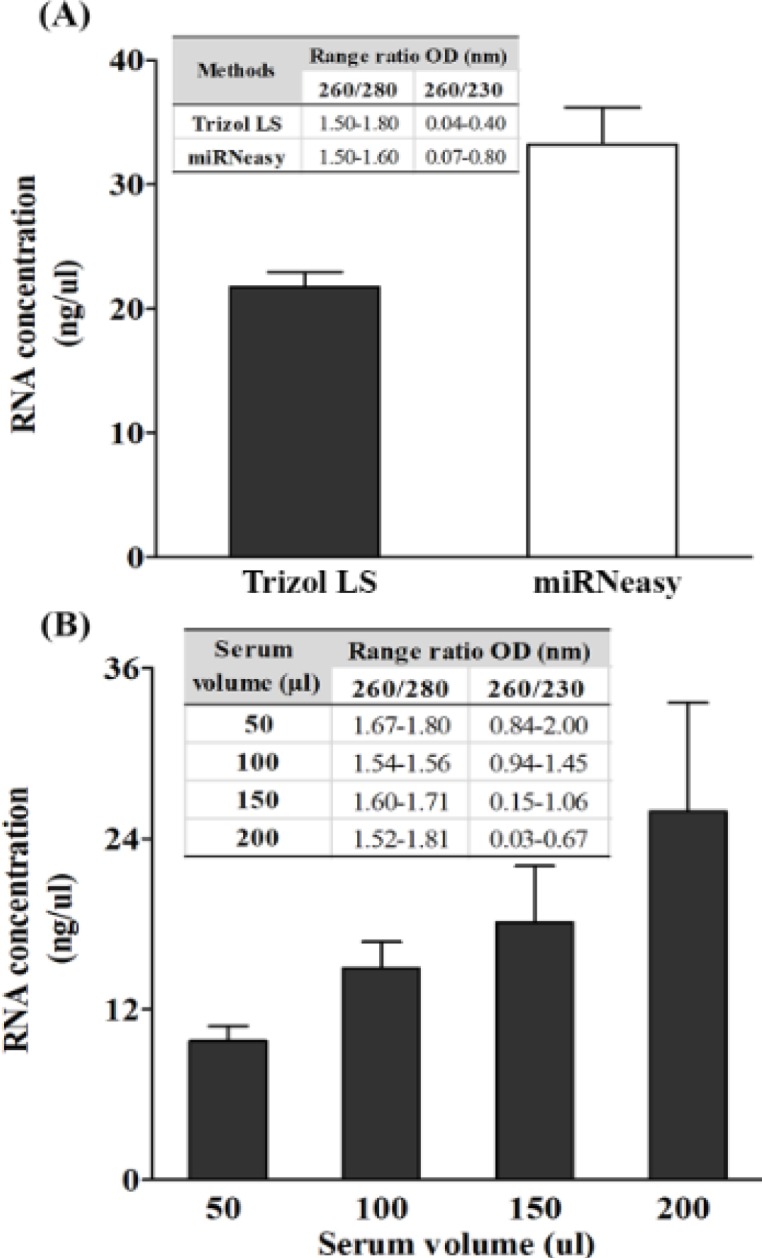
Concentration and Purity of Extracted RNAs were Isolated from Human Serum (250 μl) by; Trizol^®^LS reagent and the miRNeasy^®^mini kit (A), and human serum in various volumes (50-200 μl) by Trizol^®^LS reagent (B). Concentration and purity of total RNA were measured by NanoDrop UV-Vis spectrophotometer. The mean ± SD of three independent experiments are shown

To investigate the efficacy of Trizol^®^LS reagent compared to the commercial kit, a 250 μl volume of serum was used to isolate total RNA, according to the instruction of the manufacturer. Yields of the total RNA, by Trizol^®^LS isolation, was approximately 35% lower than that of the commercial kit. There was no difference in the ratio of absorbance at either A260/A280, or at A260/A230 amongst the two isolation methods (Figure 1A). 

**Figure 2:**
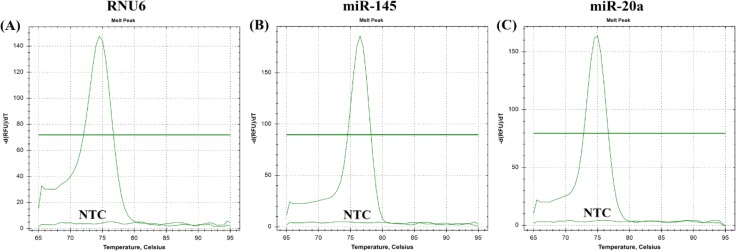
Melt Curves from qRT-PCR of Circulating Small RNAs in Serum (A) RNU6, (B) miR-145, and (C) miR-20a. NTC is no template control

The total RNA yields were improved in accordance with an increased serum volume which were 9.75±1.06 ng/μl, 14.9±1.84 ng/μl, 18.10±3.96 ng/μl, and 25.96±7.64 ng/μl for 50, 100, 150, and 200 μl volume of serum, respectively. High A260/A280 ratios were found ranging from 1.52 to 1.81 nm, whereas the A260/A230 ratio varied from 0.03 to 2.00 nm (Figure 1B). Therefore, a 150 μl volume of serum was chosen as the minimum volume for providing a sufficient yield as well as an agreeable purity for miRNA determination. By using the qRT-PCR for gene amplification, melt curves were shown in Figure 2. Single peak observed for each gene indicated that specific amplicon was generated.

*Short-term stability of serum miRNA and their respective cDNAs*

**Figure 3:**
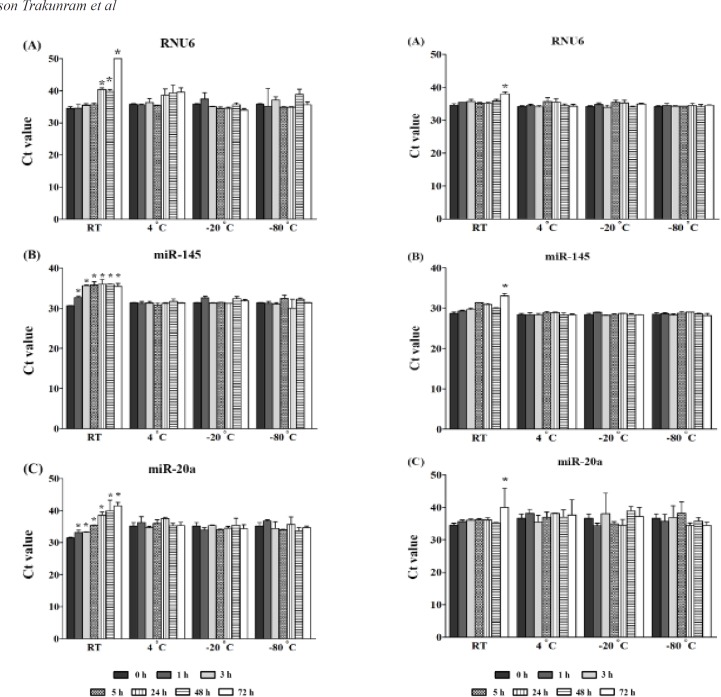
A Stability Study of Circulating Small RNAs in Serum Storage at Different Temperatures and Duration. (A) RNU6, (B) miR-145, and (C) miR-20a. The results are presented as raw Ct values. The detection of small RNAs was performed by qRT-PCR. The mean ± SD of two, or three, independent experiments are shown. An independent student t-test revealed a statistical significance (*P ≤ 0.05). Expression levels of each duration under each temperature were compared to a time of 0 hrs

**Figure 4:**
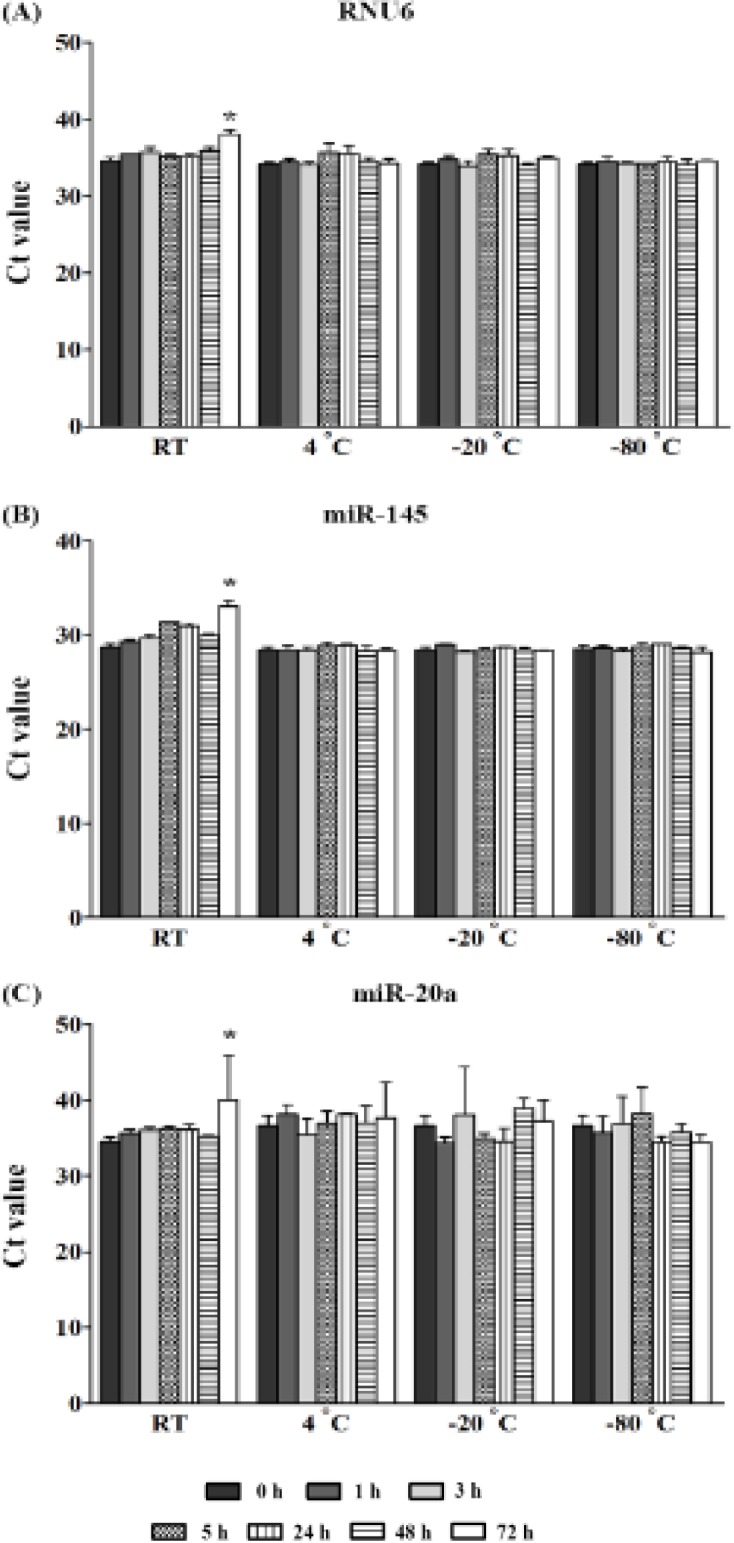
A Stability Study of Circulating RNAs in cDNA Storage at Different Temperatures as well as Duration. (A) RNU6, (B) miR-145, and (C) miR-20a. The results are presented as raw Ct values. The detection of small RNAs was performed by qRT-PCR. The mean ± SD of two, or three, independent experiments are shown. Independent student t-test revealed a statistical significance (*P ≤ 0.05). Expression levels of each duration under each temperature were compared to a time of 0 hrs

To assess the effects of short-term storage on stability, freshly isolated serum coupled with cDNA derived from RNU6, miR-145, and miR-20a were stored at 4ºC, -20ºC, and -80ºC for a 72-hrs period. We found that the amounts of all genes degraded at room temperature in both storage conditions of serum and cDNA derivatives. In serum storage conditions, the RNU6 expression was significantly decreased by; 15% at 24 hrs (P = 0.008), 13% at 48 hrs (P = 0.014), and 31% at 72 hrs (P = 0.001) as compared to 0 hrs. A significant reduction of miR-145 and miR-20a expression was also found after 1 hr ranging from a 5%-24% reduction compared to 0 hrs with a P < 0.05 (Figure 3). In the case of the storage of cDNA derivatives, the decreasing of RNU6, miR-145, and miR-20a was found after 72 hrs, which was an 11% reduction (P = 0.008), 13% reduction (P = 0.002), and 14% reduction (P = 0.04), when compared to 0 hrs, respectively (Figure 4). There was no apparent degradation of RNU6 or miRNAs at 4ºC, -20ºC, and -80ºC in different durations, for both in storage conditions of serum and cDNA derivatives. 

*Intermediate-term stability and impact of freeze-thaw cycles of respective cDNAs*

**Figure 5:**
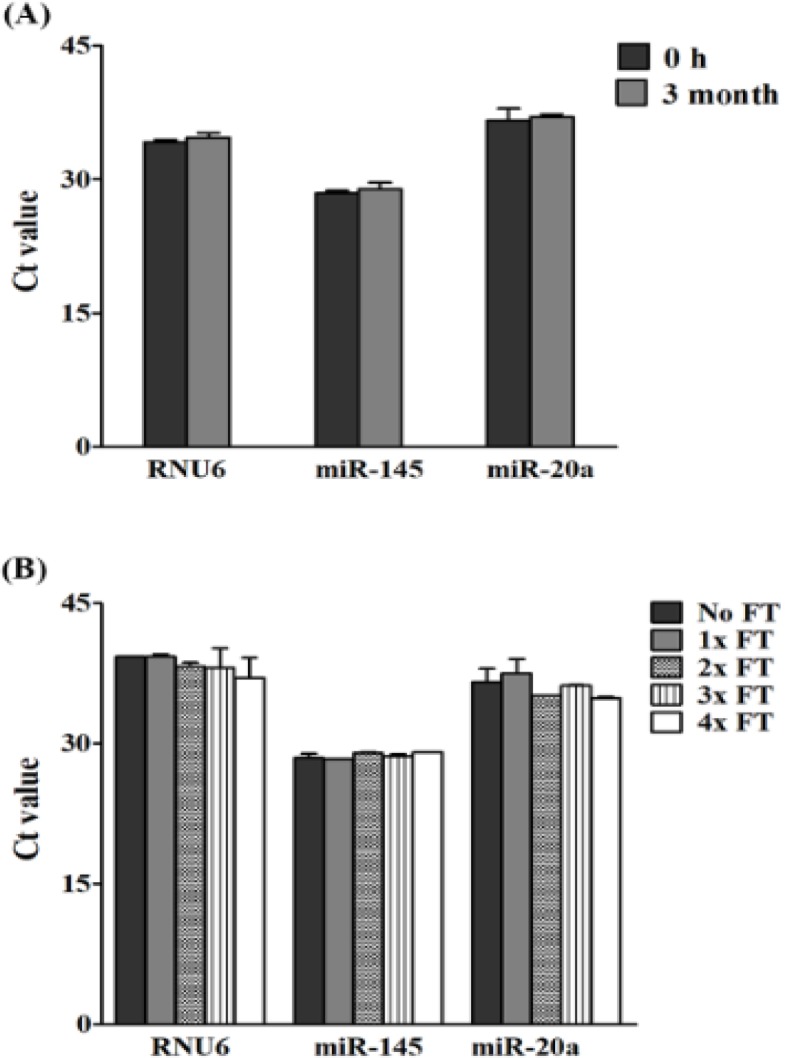
The Stability of Circulating RNAs in cDNA Storage at -20 ºC for 3 Months (A), and after, repetitive freeze-thaw (FT) cycles (B). The results are presented as raw Ct values. The detection of small RNAs was performed by qRT-PCR. The mean ± SD of three, independent experiments are shown. Independent student t-test revealed a statistical significance (*P ≤ 0.05)

There was no statistically significant difference for RNU6, miR-145, and miR-20a expression after individual cDNAs storage for 3 months at -20ºC compared to 0 hrs (Figure 5A). Similarly, all genes were found to be stable for four freeze-thaw cycles when compared to no freeze-thaw cycle (Figure 5B).

**Table 1:** Sequences of Mature miRNAs and RNU6 for qRT-PCR

Genes	Sequence (5'-3')	Length
miR-145	GTCCAGTTTTCCCAGGAATCCCT	23
miR-20a	TAAAGTGCTTATAGTGCAGGTAG	23
RNU6	GTGCTCGCTT CGGCAGCACA ATACTAAAA TTGGAACGAT ACAGAGAAGA TAGCATGGC CCCTGCGCAA GGATGACACG CAAATTCGTG AAGCGTTCCA TATTTT	107

## Discussion

As aberrant expression of miRNAs in human blood is commonly associated with various diseases, circulating miRNAs are emerging as promising biomarkers. However, the efficacy of isolated miRNAs by use of the Trizol-based method along with the stability of miRNAs in storage conditions of serum, and cDNA derivatives has been questioned. In this study, we showed that yields of total RNA, by Trizol^®^LS, were lower than those of the commercial-kit methods, whereas purity of the isolated RNAs, via both methods was similar. We have demonstrated that RNU6, miR-145, and miR-20a degraded at room temperature, but all genes were stable at 4ºC, -20ºC, and -80ºC for a 72-hrs period in both stored serum and cDNA derivatives. In addition, our data has revealed that the RNU6, miR-145, miR-20a were stable for 3 months as well as being able to resist repeated freeze-thaw cycles in stored cDNA derivatives at -20ºC. 

Theoretically, circulating miRNAs are isolated based on utilization of the Trizol-based method, or a commercial-kit method (Moldovan et al., 2014). The Trizol-based method relies on the usage of phenol and guanidinium thiocyanate, which is commonly marketed as Trizol^®^LS, for denaturing proteins and isopropyl alcohol for RNA precipitation, whereas the commercial-kit methods, such as the miRNeasy^®^mini kit, use phenol and chloroform to separate RNA and column for RNA adsorption (Moldovan et al., 2014). Some studied have shown that total RNA extracted from blood via Trizol^®^LS reagent has high yields with acceptable purity (A260/280 ratio ≥1.5) when being compared to the commercial-kit methods including; the miRNeasy^®^mini kit (Deng et al., 2005; Moret et al., 2013). However, this data is in contrast with our findings, that showed a higher yield of isolated RNAs was obtained from the miRNeasy^®^mini kit, rather than the Trizol^®^LS reagent. In addition, although our study showed an acceptable value of A260/280 ratio, in both the utilization of Trizol^®^LS reagent and miRNeasy^®^mini kit, the A260/230 ratio was low for all extraction methods. This may indicate the presence of contaminants, which absorb at 230 nm such as; phenol and guanidinium thiocyanate (El-Khoury et al., 2016), thus causing impurity of the isolated RNAs and inaccurate concentrations of input RNAs as a starting material. As a result of this, we used a fixed amount of RNA (50 ng) rather than a fixed volume of eluted RNAs for miRNA determination. Noticeably, despite the low yields and purity of Trizol^®^LS-isolated RNAs, miRNAs expression could be detected by the qRT-PCR technique in our study. These results imply that the Trizol-based method can be used to isolate the total RNA, and it is more economical to use, particularly in terms of cost rather than the commercial-kit methods for quantification of circulating miRNAs.

For circulating miRNAs, intensive studies have provided evidences that the miRNAs are released from cells into the circulation from several types including; apoptotic body-enclosed miRNA, shedding vesicles/exosomes packaged miRNA, HDL-associated miRNA, and vesicles-free miRNAs (Turchinovich et al., 2012; Sohel, 2016). For the latter type, they represent the majority by approximately 90-95% of all miRNAs, and it has remarkable stability, because of their stabilizing by AGO proteins (Turchinovich et al., 2011; Winter and Diederichs, 2011).Several studies have revealed that the circulating miRNAs are stable in different conditions. Chen et al., (2008) has demonstrated that miRNAs in both extracted serum RNAs and serum without RNA extraction are resistant to RNaseA digestion, low/high pH, and 10 freeze-thaw cycles at -80°C (Chen et al., 2008; Mitchell et al., 2008). Likewise, miR-451a and miR-23a in unhemolyzed blood samples, kept at room temperature, and at 4°C are stable for at least 8 hrs (Wu et al., 2016) as well as storage of plasma at -80˚C for 9 months having no significant effect on miR-21 and miR-29b levels (Glinge et al., 2017). Moreover, Grasedieck et al., (2012) have shown that miRNAs, from serum, are stable at -20˚C and -80˚C for 10 days (short-term storage), 20 months (intermediate-term storage), and at least 4 years (long-term storage). Similar to our results, we found that miRNAs and RNU6 were stable at 4°C, -20°C, and -80°C. However, instability of these genes was shown at room temperature in both stored serum and cDNA derivatives.

Indeed, there are an increasing amount of studies showing that the stability of miRNAs is varied upon prolong incubation and in different storage conditions, depending on the types of miRNAs. Glinge et al., (2017) found that miR-21 and miR-1 are stable for at least 4 days in EDTA-treated whole blood, whereas only miR-1 significantly degraded within 4 days in serum, separator-treated, whole blood. Some studies have shown that the degradation of miR-1 and miR-122 expeditiously occurred within 3 hrs, whereas other miRNAs declined only slightly within the initial 5 hrs, after serum storage at room temperature (Koberle et al., 2013). In addition, instability of miRNAs is also observed after repeated freeze-thaw cycles for miR-24, miR-93, miR-223, miR-451, and miR-21 when plasma or serum are kept at -20˚C and -80˚C (Grasedieck et al., 2012; Glinge et al., 2017). There are several reasons to explain the inconsistency in these results. One explanation is the sample type differences, or sample processing protocols. For example, the presence of lithium-heparin as anticoagulants, and mechanical disturbances such as; the shaking for plasma isolation results in degradation of miRNAs (Glinge et al., 2017). In recent years, Kakimoto et al., (2016) have demonstrated that the miRNA stability is also depended on the GC content in their sequence, by which the miRNAs containing a GC content of less than 40% are more degraded than those of GC-rich miRNAs. These evidences may imply that storage of whole blood, serum, and in particularly plasma, may not be a proper source for miRNA determination. 

For quantitative, PCR studies of miRNAs and other small RNAs, the isolated total RNA is polyadenylated by poly(A)polymerase, and reverse transcribed into cDNA using oligo-dT primers. By these processes, we hypothesized that the length of miRNAs are extended, and in turn this may change the GC content, resulting in increasing of miRNA stability. This hypothesis is in accordance with our results, showing that RNU6 and miRNAs were more stable in stored cDNA derivatives than stored serum containing a short length of miRNAs at room temperature. Therefore, in this present study, we proposed that the storage of miRNA-derived cDNAs may be an alternative choice, so as to avoid this stability effect. 

In conclusion, the results of this study have demonstrated that the Trizol-based method is efficient, and economical for the use in quantification of circulating miRNAs. The RNU6, miR-145, and miR-20a were stable at 4ºC, -20ºC, and -80ºC for a 72-hrs period, except at room temperature in both stored serum and cDNA conditions. Additionally, in cDNA storage, all genes were stable for 3 months, as well as being able to resist repeated freeze-thaw cycles at -20ºC. Finally, for miRNA quantification, we suggested that whole blood samples should be processed into serum within 1 hr, at room temperature, and that separated serum can be kept at 4ºC, -20ºC, and -80ºC for at least 72 hrs. After which, total RNA should be isolated, and immediately converted to cDNA for storage at -20ºC for at least 3 months before analysis.

## Statement conflict of Interest

The authors declare that they have no competing interests.
